# Childhood trauma and suicidal behavior risk: perceived burdensomeness and negative emotional reactivity in a cross-sectional study

**DOI:** 10.3389/fpsyg.2026.1923772

**Published:** 2026-08-04

**Authors:** Tolga Köskün

**Affiliations:** Department of Psychology, Aydın Adnan Menderes University, Aydın, Türkiye

**Keywords:** childhood trauma, negative emotional reactivity, perceived burdensomeness, suicidal behavior risk, thwarted belongingness

## Abstract

**Introduction:**

Childhood traumatic experiences are associated with a wide range of psychological difficulties in adulthood and have also been associated with an increased risk of suicidal thoughts and behaviors. However, the interpersonal and emotional factors that may be relevant to the association between childhood trauma and suicidal behavior risk remain insufficiently understood. Grounded in the Interpersonal-Psychological Theory of Suicide, this cross-sectional study examined perceived burdensomeness and thwarted belongingness as parallel interpersonal factors linking childhood trauma to suicidal behavior risk. It further examined whether the association between these interpersonal factors and suicidal behavior risk differed according to levels of negative emotional reactivity.

**Methods:**

Participants were 340 community adults in Turkey aged 18–63 years (*M* = 23.43, *SD* = 5.83) who completed an anonymous online survey including self-report measures of childhood trauma, interpersonal vulnerability factors, negative emotional reactivity, and suicidal behavior risk. Descriptive statistics, bivariate correlations, and a parallel conditional indirect association model were tested, controlling for age and gender.

**Results:**

Childhood trauma was positively associated with suicidal behavior risk, perceived burdensomeness, thwarted belongingness, and negative emotional reactivity. When both interpersonal variables were examined simultaneously, only perceived burdensomeness was significantly associated with suicidal behavior risk. Negative emotional reactivity moderated the perceived burdensomeness–suicidal behavior risk association, with the association being stronger at mean and high levels of negative emotional reactivity. The corresponding statistical indirect association through perceived burdensomeness was significant at mean and high, but not low, levels of negative emotional reactivity. The indirect association through thwarted belongingness was not significant.

**Discussion:**

Perceived burdensomeness emerged as the most relevant interpersonal correlate of suicidal behavior risk in the context of childhood trauma, particularly among individuals with elevated negative emotional reactivity. Given the cross-sectional self-report design, the results should be interpreted as conditional statistical associations rather than evidence of temporal or causal pathways. These findings highlight the importance of considering interpersonal and emotional vulnerability factors together when examining suicidal behavior risk among individuals with childhood trauma histories.

## 1 Introduction

Childhood trauma is a major developmental and public health concern with enduring implications for psychological functioning, interpersonal relationships, and emotion regulation (Dye, 2018; McKay et al., 2021). It commonly includes physical, emotional, and sexual abuse, as well as physical and emotional neglect (Norman et al., 2012). A substantial body of evidence has linked childhood trauma to suicidal ideation, suicide planning, and suicide attempts across both general and clinical populations (Angelakis et al., 2020; Baldini et al., 2025; Liu et al., 2017; Zatti et al., 2017). Suicide itself remains a major public health problem worldwide. According to the World Health Organization (2025), more than 720,000 people die by suicide each year, suicide is the third leading cause of death among individuals aged 15–29 years, and for every death by suicide, there are approximately 20 suicide attempts.

Meta-analytic evidence indicates that childhood abuse is associated with a two- to threefold increase in suicide attempt risk and is also significantly related to suicidal ideation (Angelakis et al., 2019). Moreover, exposure to multiple forms of childhood trauma may be associated with particularly elevated suicidal behavior risk in adulthood (Angelakis et al., 2020; Liu et al., 2017). Although the association between childhood trauma and suicidal behavior risk is well established, the interpersonal and emotional vulnerability processes that may help account for this association remain less clearly understood. Examining these processes may be important for understanding the interpersonal and emotional contexts in which childhood trauma is associated with later suicidal behavior risk and for informing trauma-informed suicide prevention efforts.

The Interpersonal-Psychological Theory of Suicide (IPTS; Joiner, 2005; Van Orden et al., 2010) is a theory of suicidal thoughts and behaviors that provides a useful framework for conceptualizing proximal interpersonal vulnerabilities associated with suicidal behavior risk. In the present study, interpersonal vulnerabilities refer to perceived burdensomeness and thwarted belongingness, two interpersonal constructs central to the IPTS. Perceived burdensomeness refers to the perception that one is a burden on others, whereas thwarted belongingness refers to social disconnection, a lack of meaningful interpersonal relationships, and an unmet need to belong. Although childhood trauma is not a central construct of the IPTS, it can be theoretically linked to the interpersonal constructs proposed by the theory. Early experiences of abuse and neglect may constitute a distal developmental context associated with perceived burdensomeness and thwarted belongingness, which the IPTS identifies as proximal interpersonal states relevant to suicidal desire. The IPTS also includes acquired capability for suicide, which is thought to facilitate the transition from suicidal desire to suicidal behavior. Given the focus of the present study on interpersonal vulnerabilities in the association between childhood trauma and suicidal behavior risk, perceived burdensomeness and thwarted belongingness were examined, whereas acquired capability was beyond the scope of the current model.

Theoretical accounts suggest that experiences of abuse and neglect may contribute to feelings of being unloved, unwanted, and worthless, which may be reflected in perceived burdensomeness and thwarted belongingness (Joiner, 2005; Joiner et al., 2007; Van Orden et al., 2010). Empirical findings support these theoretical links. Among hospitalized patients with depression, emotional abuse was positively associated with perceived burdensomeness and thwarted belongingness (Schönfelder et al., 2019), whereas physical, emotional, and sexual abuse were associated with higher perceived burdensomeness in psychiatric patients (Schönfelder et al., 2021). Similarly, perceived burdensomeness and thwarted belongingness have been implicated in the association between childhood trauma and suicidal behavior risk among individuals with mood disorders, university students, and non-clinical young adults (Lee et al., 2025; Smith et al., 2018; Spínola et al., 2022). Together, these findings suggest that perceived burdensomeness and thwarted belongingness may represent proximal interpersonal vulnerability processes involved in the association between childhood trauma and suicidal behavior risk. However, less is known about whether emotional vulnerability factors shape the strength of the associations between these interpersonal vulnerabilities and suicidal behavior risk.

Negative emotional reactivity may be one such vulnerability factor. Emotional reactivity refers to individual differences in the ease of activation, intensity, and duration of emotional responses (Nock et al., 2008; Preece et al., 2019). From a transdiagnostic perspective, heightened emotional reactivity can be situated within broader models of emotion regulation and affective disturbance (Gross, 2014; Gross and Jazaieri, 2014). Although previous suicide-related studies have often examined emotion reactivity as a broad construct, the present study focused specifically on negative emotional reactivity (Becerra and Campitelli, 2013; Preece et al., 2019). This focus is appropriate because the measure used in the present study assesses reactivity to negative emotions and because negative emotional reactivity is conceptually aligned with the aversive emotional and interpersonal experiences reflected in childhood trauma, perceived burdensomeness, thwarted belongingness, and suicidal behavior risk. Individuals with elevated negative emotional reactivity may experience more intense and persistent affective responses to stress, which may make interpersonal distress more salient and difficult to regulate. Accordingly, negative emotional reactivity may represent an affective vulnerability context in which perceived burdensomeness and thwarted belongingness are more strongly associated with suicidal behavior risk. This perspective is consistent with theoretical models suggesting that heightened emotional reactivity may increase vulnerability to stress-related suicidal and self-harming responses (Brodsky, 2016; Linehan et al., 2015; O'Connor and Kirtley, 2018; van Heeringen and Mann, 2014). Empirical findings also indicate that emotion reactivity is associated with suicidal ideation and a history of suicide attempts (DeCou and Lynch, 2019; Shapero et al., 2019; Wang et al., 2021; Wu et al., 2023). Thus, negative emotional reactivity may be particularly relevant for understanding the emotional conditions under which interpersonal vulnerabilities are more strongly associated with suicidal behavior risk. However, this possibility remains less clearly understood in the context of childhood trauma and interpersonal vulnerability processes.

**Figure 1:**
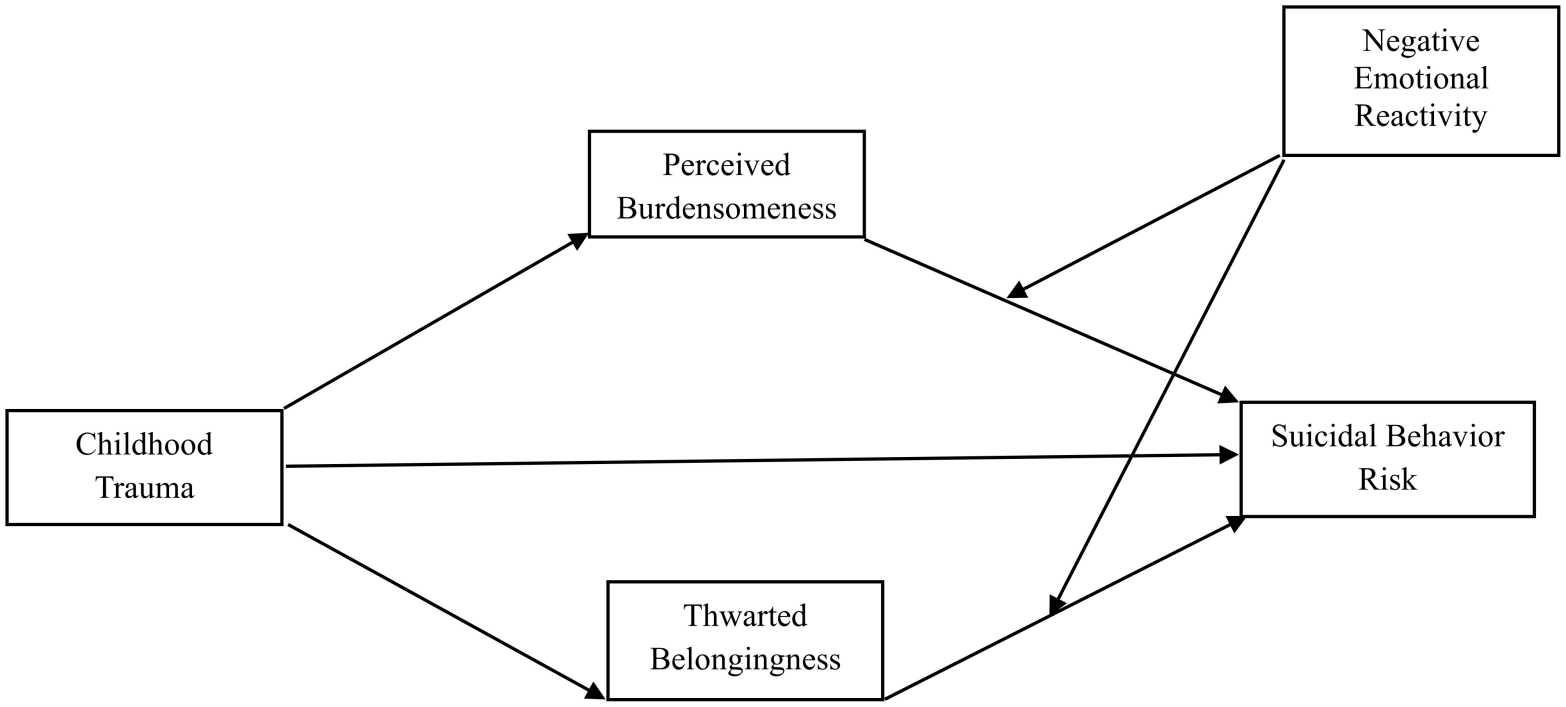
Conceptual model of the conditional process analysis.

Building on this literature, the present cross-sectional study aimed to investigate the association between childhood trauma and suicidal behavior risk within a theoretical framework that conceptualizes childhood trauma as a distal developmental context and perceived burdensomeness and thwarted belongingness as more proximal interpersonal vulnerabilities. Specifically, it examined whether perceived burdensomeness and thwarted belongingness were involved in this association and whether negative emotional reactivity moderated the associations between these interpersonal vulnerabilities and suicidal behavior risk. Perceived burdensomeness and thwarted belongingness were examined simultaneously in a parallel model to evaluate their unique contributions while accounting for their shared variance. It was expected that childhood trauma would be positively associated with suicidal behavior risk, perceived burdensomeness, and thwarted belongingness. It was also expected that perceived burdensomeness and thwarted belongingness would be positively associated with suicidal behavior risk, and that these associations, as well as the corresponding indirect associations, would be stronger among individuals with higher levels of negative emotional reactivity. The conceptual model is shown in Figure 1.

## 2 Materials and methods

### 2.1 Participants

The sample consisted of 340 community adults in Türkiye, recruited through convenience sampling. Participants were aged between 18 and 63 years *(M* = 23.43, *SD* = 5.83), and 75.6% were women. Although the age range was broad, the sample was concentrated in young adulthood, with 85.0% of participants aged between 18 and 25 years. All participants were native Turkish speakers. Regarding suicide-related indicators, 5.3% of participants reported current suicidal ideation, 12.1% reported at least one lifetime suicide attempt, and 17.0% reported a lifetime psychiatric diagnosis.

### 2.2 Measures

#### 2.2.1 Childhood trauma

The Childhood Trauma Questionnaire–Short Form (CTQ-SF; Bernstein et al., 2003; Turkish version: Sar et al., 2012) was used to assess childhood abuse and neglect. This 28-item self-report measure assesses five types of childhood trauma: emotional abuse, physical abuse, sexual abuse, emotional neglect, and physical neglect, as well as a minimization/denial validity scale. Items are rated on a 5-point Likert scale ranging from 1 (never) to 5 (very often). Higher scores indicate greater childhood trauma exposure. In the Turkish adaptation study, internal consistency coefficients ranged from 0.71 to 0.90 (Sar et al., 2012). In the present study, the total childhood trauma score was computed by summing the five maltreatment subscale scores„ and Cronbach's alpha was 0.85.

#### 2.2.2 Suicidal behavior risk

The Suicidal Behaviors Questionnaire (SBQ; Linehan and Nielsen, 1981; Turkish version: Bayam et al., 1995) was used to assess suicidal behavior risk. This four-item self-report measure assesses lifetime suicidal ideation and suicide attempts, frequency of suicidal ideation over the past year, communication of suicidal intent, and the perceived likelihood of future suicidal behavior. Total scores range from 0 to 14, with higher scores indicating greater suicidal behavior risk. The Turkish adaptation reported an internal consistency coefficient of 0.73 (Bayam et al., 1995). In the present study, Cronbach's alpha was 0.76.

#### 2.2.3 Perceived burdensomeness and thwarted belongingness

The Interpersonal Needs Questionnaire-10 (INQ-10; Bryan, 2011; Turkish version: Eskin et al., 2020) was used to assess perceived burdensomeness and thwarted belongingness. This 10-item self-report measure includes two subscales: perceived burdensomeness and thwarted belongingness. Each subscale consists of five items rated on a 7-point Likert scale ranging from 1 (strongly disagree) to 7 (strongly agree). Higher scores indicate greater perceived burdensomeness and thwarted belongingness. In the Turkish adaptation study, internal consistency coefficients were 0.90 for perceived burdensomeness and 0.79 for thwarted belongingness (Eskin et al., 2020). In the present study, subscale scores were used, and Cronbach's alpha coefficients were 0.91 for perceived burdensomeness and 0.71 for thwarted belongingness.

#### 2.2.4 Negative emotional reactivity

The Perth Emotional Reactivity Scale–Short Form (PERS-S; Preece et al., 2019; Turkish version: Gökdağ et al., 2024) was used to assess negative emotional reactivity. This 18-item self-report measure assesses individual differences in positive and negative emotional reactivity. Items are rated on a 5-point Likert scale ranging from 1 (very unlike me) to 5 (very like me). The scale includes six lower-order subscales and two higher-order composite scores: general positive reactivity and general negative reactivity. In the present study, the general negative reactivity composite score was used, which comprises negative activation, negative intensity, and negative duration. Negative emotional reactivity was selected a priori to align with the study's focus on risk-related emotional processes; therefore, positive emotional reactivity was not included in the analyses. Higher scores indicate greater negative emotional reactivity. In the Turkish adaptation study, internal consistency coefficients ranged from 0.72 to 0.85 for the PERS-S subscales, and the coefficient for general negative reactivity was 0.90 (Gökdağ et al., 2024). Cronbach's alpha for the general negative reactivity composite score was 0.91 in the present study.

#### 2.2.5 Demographic and suicide-related characteristics

A demographic information form was used to collect information on participants' age, gender, and suicide-related indicators, including current suicidal ideation, lifetime suicide attempt, and lifetime psychiatric diagnosis. These indicators were assessed using binary yes/no questions.

### 2.3 Procedures

The study protocol was approved by the Aydin Adnan Menderes University Social and Human Sciences Ethics Committee (Protocol No: 08/14). Participants were recruited through convenience sampling, and the online survey link was distributed via email and social media platforms, including [Instagram, WhatsApp, etc.]. Before beginning the survey, participants were presented with an online informed consent form describing the study purpose, procedures, voluntary nature of participation, and anonymity of responses. Participants were informed that they could discontinue participation at any time, and could stop completing the survey if they experienced discomfort. Only those who provided electronic informed consent proceeded to complete the questionnaires. No personally identifying information was collected. Given the sensitive nature of the suicide-related questions, support information was provided at the end of the survey, including guidance to seek professional support from psychiatry departments of public or university hospitals, family physicians, university health units, and psychological counseling centers when available. Due to the anonymous online survey design, individualized follow-up or tiered risk-response procedures could not be implemented.

### 2.4 Data analysis

Data were analyzed using IBM SPSS Statistics 27.0 and the PROCESS macro (Hayes, 2013). Descriptive statistics and bivariate correlations were computed. The results of Harman's single-factor test showed that the first factor accounted for 25.99% of the variance, suggesting that common method variance was not a dominant concern (Podsakoff et al., 2003). A parallel moderated mediation model was tested using PROCESS Model 14, with childhood trauma as the predictor, suicidal behavior risk as the outcome, perceived burdensomeness and thwarted belongingness as simultaneous mediators, and negative emotional reactivity as the moderator of the mediator-to-outcome paths. Age and gender were included as covariates. Continuous variables involved in interaction terms were mean-centered. Conditional indirect associations were estimated using 5,000 bootstrap samples and 95% bootstrap confidence intervals at low (−1 SD), mean, and high (+1 SD) levels of negative emotional reactivity. The index of moderated mediation was examined for each mediator, and effects were considered statistically significant when the 95% bootstrap confidence interval did not include zero. A sensitivity power analysis was conducted using G^*^Power 3.1 to evaluate sample-size adequacy for the regression-based analyses. For the most complex regression model, which included eight predictors, the available sample size of 340 provided 80% power at α = 0.05 to detect an effect size of approximately *f*^2^ = 0.045 or larger. Given the cross-sectional design, the PROCESS analyses were used to estimate conditional indirect associations and were not interpreted as evidence of temporal or causal mediation.

## 3 Results

### 3.1 Descriptive and correlational statistics

Descriptive statistics and Pearson correlation coefficients for the study variables are presented in Table 1. Childhood trauma was positively correlated with perceived burdensomeness, thwarted belongingness, negative emotional reactivity, and suicidal behavior risk. Suicidal behavior risk was also positively correlated with perceived burdensomeness, thwarted belongingness, and negative emotional reactivity. Negative emotional reactivity was positively correlated with both perceived burdensomeness and thwarted belongingness. Among the covariates, only gender was significantly associated with negative emotional reactivity, with higher scores among women.

**Table 1:** Means (M), standard deviations (SD), and correlations among study variables.

Variable	1	2	3	4	5	6	7	*M*	*SD*
1. Age	-	0.05	0.03	−0.09	−0.04	−0.06	−0.05	23.43	5.83
2. Gender		-	−0.02	0.09	−0.03	0.18^**^	0.02	-	-
3. Childhood trauma			-	0.33^**^	0.32^**^	0.14^*^	0.48^**^	40.36	13.64
4. Perceived burdensomeness				-	0.49^**^	0.42^**^	0.54^**^	11.49	6.49
5. Thwarted belongingness					-	0.35^**^	0.34^**^	17.81	6.08
6. Negative Emotional Reactivity						-	0.34^**^	30.93	7.02
7. Suicidal Behavior Risk							-	2.40	2.47

Childhood trauma was assessed using the CTQ total score; suicidal behavior risk was assessed using the SBQ total score. Gender was dummy-coded as 0 = men and 1 = women; correlations involving gender represent point-biserial correlations. ^*^p < 0.05 ^**^p < 0.01.

### 3.2 Parallel moderated mediation analysis

**Figure 2:**
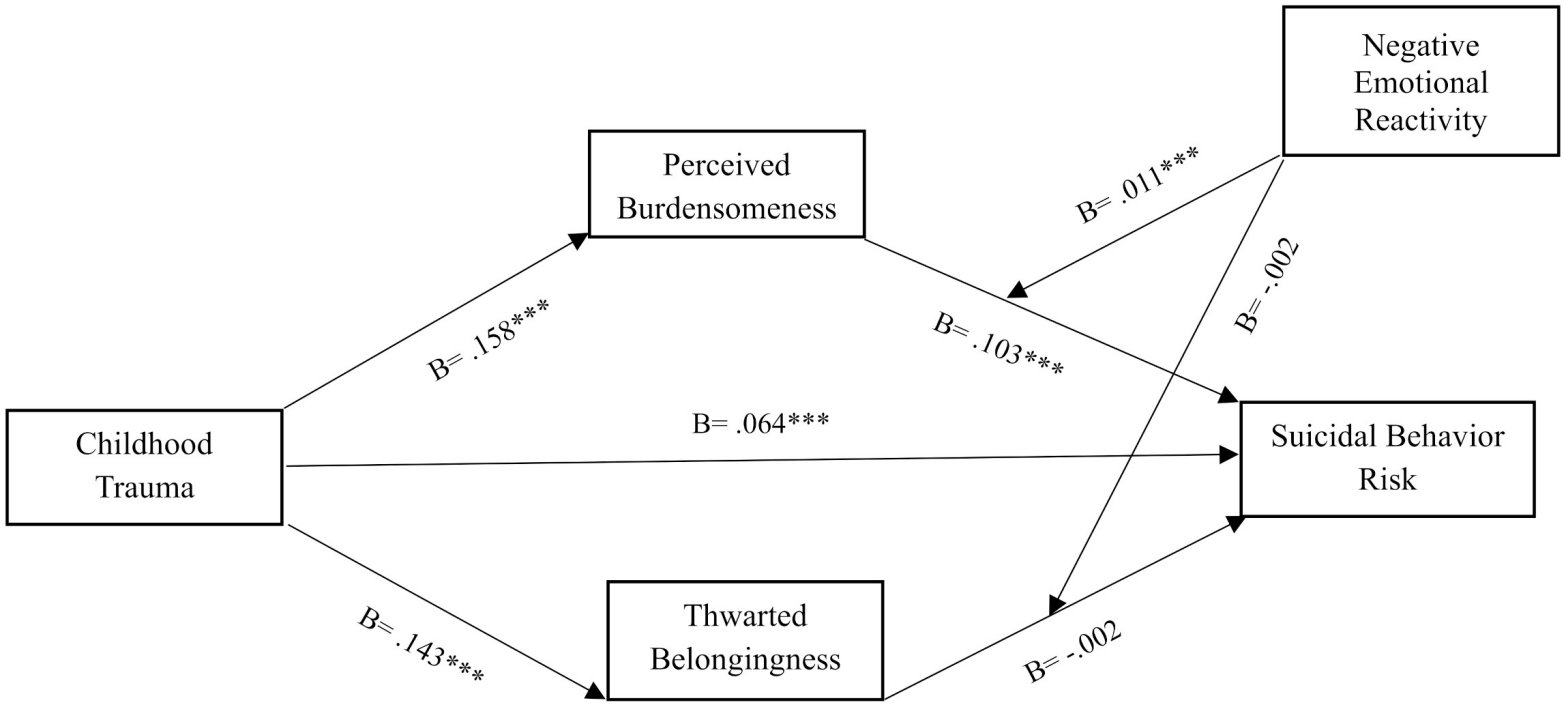
Statistical diagram of the parallel moderated mediation model. Values are unstandardized regression coefficients. **p < 0.001. Non-significant paths are shown without asterisks.

A parallel moderated mediation model was tested to examine conditional indirect associations between childhood trauma and suicidal behavior risk through perceived burdensomeness and thwarted belongingness. Perceived burdensomeness and thwarted belongingness were entered simultaneously as mediators, and negative emotional reactivity was specified as a moderator of the associations between these interpersonal vulnerabilities and suicidal behavior risk. The results are presented in Table 2 and visually summarized in Figure 2.

**Table 2:** Results of the parallel moderated mediation model including perceived burdensomeness and thwarted belongingness.

	Antecedent models									
	PB					TB				
Antecedents				95% CI					95% CI	
	*B*	*SE*	*t*	Lower	Upper	*B*	*SE*	*t*	Lower	Upper
CT	0.158	0.024	6.53	0.1106	0.2059	0.143	0.023	6.21	0.0977	0.1883
**Outcome model: suicidal behavior risk**										
				**95% CI**						
**Antecedents**	***B***	***SE***	***t***	**Lower**	**Upper**					
CT	0.064	0.008	7.98	0.0483	0.0800					
PB	0.103	0.021	4.77	0.0605	0.1455					
TB	−0.002	0.019	−0.12	−0.0418	0.0367					
PB x NER	0.011	0.002	4.01	0.0056	0.0165					
TB x NER	−0.002	0.002	−0.88	−0.0072	0.0027					
Constant	−0.333	0.606	−0.55	−1.526	0.8584				
	*R*=0.66, *R*^2^ =0.45, *F*_(8, 331)_ = 33.60, *p < 0*.001									
**Conditional indirect effects of CT on SBR via PB at levels of NER**										
			**95% Bootstrap CI**							
**Level of NER**	***B***	***BootSE***	**Lower**	**Upper**						
Low (−1 SD)	0.003	0.006	−0.0086	0.0181						
Average (Mean)	0.016	0.005	0.0075	0.0277						
High (+1 SD)	0.028	0.007	0.0159	0.0451						
**Conditional indirect effects of CT on SBR via TB at levels of NER**										
			**95% Bootstrap CI**							
**Level of NER**	***B***	***BootSE***	**Lower**	**Upper**						
Low (−1 SD)	0.002	0.002	−0.0037	0.0077						
Average (Mean)	−0.0003	0.002	−0.0065	0.0053						
High (+1 SD)	−0.002	0.004	−0.0125	0.0062						
**Index of moderated mediation**										
**Pathway**	**Index**	***BootSE***	**Lower**	**Upper**						
Via perceived burdensomeness	0.0018	0.0007	0.0006	0.0033						
Via thwarted belongingness	−0.0003	0.0004	−0.0011	0.0004						

CT, Childhood Trauma; PB, Perceived Burdensomeness; TB, Thwarted Belongingness; NER, Negative Emotional Reactivity; SBR, Suicidal Behavior Risk; CI, Confidence Interval.

**Figure 3:**
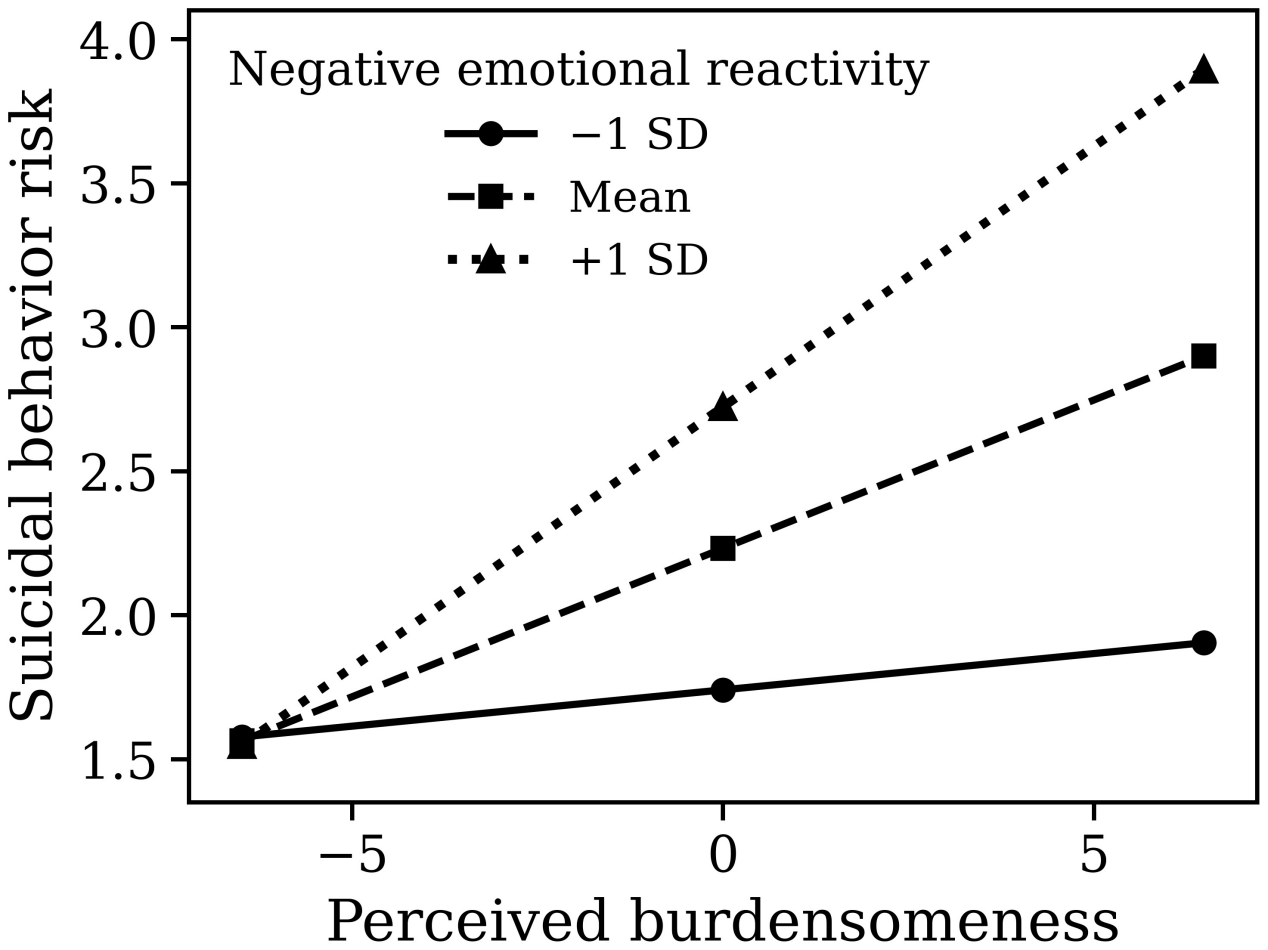
Simple slopes of the association between perceived burdensomeness and suicidal behavior risk at low, mean, and high levels of negative emotional reactivity. Low and high levels represent −1 SD and +1 SD from the mean of negative emotional reactivity, respectively.

Controlling for age and gender, childhood trauma was positively associated with both perceived burdensomeness (*B* = 0.158, *SE* = 0.024, *p* < 0.001) and thwarted belongingness (*B* = 0.143, *SE* = 0.023, *p* < 0.001). In the outcome model, childhood trauma (*B* = 0.064, *SE* = 0.008, *p* < 0.001) and perceived burdensomeness (*B* = 0.103, *SE* = 0.021, *p* < 0.001) were positively associated with suicidal behavior risk, whereas thwarted belongingness was not (*B* = −0.002, *SE* = 0.019, *p* = 0.898). The model explained 45% of the variance in suicidal behavior risk. The interaction between perceived burdensomeness and negative emotional reactivity was significant (*B* = 0.011, *SE* = 0.002, *p* < 0.001). Simple slope analyses indicated that the association between perceived burdensomeness and suicidal behavior risk was significant at mean and high, but not low, levels of negative emotional reactivity. This interaction is illustrated in Figure 3. In contrast, the interaction between thwarted belongingness and negative emotional reactivity was not significant (*B* = −0.002, *SE* = 0.002, *p* = 0.374).

As shown in Table 2, the conditional indirect association involving perceived burdensomeness was not significant at low levels of negative emotional reactivity, but was significant at the mean (*B* = 0.016, *BootSE* = 0.005, 95% bootstrap CI [0.0075, 0.0277]) and high levels (*B* = 0.028, *BootSE* = 0.007, 95% bootstrap CI [0.0159, 0.0451]). The corresponding conditional indirect associations involving thwarted belongingness were not significant. The index of moderated mediation was significant for perceived burdensomeness (index = 0.0018, *BootSE* = 0.0007, 95% bootstrap CI [0.0006, 0.0033]), whereas the index for thwarted belongingness was not significant (index = −0.0003, *BootSE* = 0.0004, 95% bootstrap CI [−0.0011, 0.0004]). Thus, the moderated indirect association between childhood trauma and suicidal behavior risk was observed only in relation to perceived burdensomeness.

## 4 Discussion

The present cross-sectional study examined the association between childhood trauma and suicidal behavior risk in relation to perceived burdensomeness and thwarted belongingness, and whether the corresponding indirect associations varied as a function of negative emotional reactivity. Overall, the findings were partially consistent with the proposed model. Childhood trauma was positively associated with suicidal behavior risk and with both interpersonal vulnerabilities. However, when perceived burdensomeness and thwarted belongingness were examined simultaneously, only perceived burdensomeness was uniquely associated with suicidal behavior risk and showed a significant conditional indirect association. This conditional indirect association was observed at mean and high levels of negative emotional reactivity, whereas the corresponding association involving thwarted belongingness was not significant.

### 4.1 Childhood trauma, interpersonal vulnerabilities, and suicidal behavior risk

Consistent with the interpersonal focus of the present study, childhood trauma was positively associated not only with suicidal behavior risk but also with perceived burdensomeness and thwarted belongingness. This pattern is consistent with previous research showing that childhood trauma is related to suicidal ideation, suicide attempts, and broader suicide risk indicators across clinical and non-clinical samples (Baldini et al., 2025; Liu et al., 2017). It also aligns with studies linking childhood trauma to interpersonal vulnerabilities, including perceived burdensomeness and thwarted belongingness (Allbaugh et al., 2017; Lee et al., 2025; Schönfelder et al., 2019; Smith et al., 2018). From the perspective of the IPTS, experiences of childhood trauma may be associated with feelings of being unloved, unwanted, and worthless, which may be reflected in perceptions of being a burden on others or lacking meaningful interpersonal connection (Joiner, 2005; Joiner et al., 2007; Van Orden et al., 2010). The association between childhood trauma and thwarted belongingness may also be understood in relation to disruptions in interpersonal trust and connectedness. Janoff-Bulman's assumptive world theory suggests that traumatic experiences may disrupt basic assumptions about safety, meaning, and the trustworthiness of others (Janoff-Bulman, 2006). Such disruptions may be associated with social withdrawal, difficulties in forming close bonds, and a diminished sense of interpersonal closeness (Schuler and Boals, 2016), which are central to experiences of thwarted belongingness. Taken together, these findings suggest that childhood trauma is associated with interpersonal vulnerabilities involving self-worth, perceived value to others, interpersonal trust, and relational connectedness.

### 4.2 Perceived burdensomeness as a specific interpersonal correlate of suicidal behavior risk

When perceived burdensomeness and thwarted belongingness were examined simultaneously, only perceived burdensomeness was uniquely associated with suicidal behavior risk. This finding is consistent with the IPTS and with evidence suggesting that perceived burdensomeness is one of the most robust interpersonal correlates of suicidal thoughts and behaviors (Chu et al., 2017; Ma et al., 2016). Perceived burdensomeness may be particularly relevant because it involves a self-focused and socially evaluative belief that one is a burden or liability to others. Individuals with higher perceived burdensomeness may believe that they make the lives of significant others more difficult, impose emotional or practical strain on others, or that others would be better off without them. These burden-related self-evaluations are closely aligned with the theoretical meaning of perceived burdensomeness and may help account for why this construct showed a more specific association with suicidal behavior risk in the present cross-sectional model.

In contrast, thwarted belongingness was not uniquely associated with suicidal behavior risk when perceived burdensomeness was included in the same model. This finding should be interpreted in the context of the simultaneous model. Although thwarted belongingness was associated with suicidal behavior risk at the bivariate level, it did not account for unique variance once perceived burdensomeness was taken into account. Meta-analytic evidence indicates that perceived burdensomeness and thwarted belongingness are empirically related but not redundant, and that perceived burdensomeness shows stronger associations with suicidal ideation and suicide risk than thwarted belongingness (Chu et al., 2017). Similarly, a systematic review of IPTS studies suggested that perceived burdensomeness was the most consistently supported interpersonal correlate of suicidal ideation, whereas findings for thwarted belongingness were more mixed (Ma et al., 2016). This does not imply that thwarted belongingness is irrelevant to suicide-related risk. Rather, the present pattern may reflect both the empirical overlap between these two interpersonal constructs and the more specific relevance of burden-related self-evaluations in the present model. Conceptually, the two interpersonal constructs are distinct within the IPTS (Joiner, 2005; Van Orden et al., 2010). Perceived burdensomeness directly involves beliefs about being a burden, source of strain, or liability to significant others, whereas thwarted belongingness reflects broader experiences of disconnection, lack of belonging, reduced closeness, and social alienation. Although these experiences are theoretically relevant to suicide-related risk, they may be less specifically associated with suicidal behavior risk than burden-related beliefs when both interpersonal constructs are considered simultaneously. Thus, perceived burdensomeness appeared to show a more specific association with suicidal behavior risk in the present model. However, given the non-clinical community sample and cross-sectional design, this interpretation should be considered tentative and examined further in clinical, high-risk, and longitudinal samples.

### 4.3 Negative emotional reactivity and conditional indirect associations

The findings showed a conditional pattern specific to perceived burdensomeness. Negative emotional reactivity moderated the association between perceived burdensomeness and suicidal behavior risk, and the conditional indirect association involving perceived burdensomeness was significant at mean and high levels of negative emotional reactivity, but not at low levels. In contrast, the corresponding conditional indirect association involving thwarted belongingness was not significant. This pattern suggests that negative emotional reactivity may represent an affective vulnerability context in which burden-related self-evaluations are more strongly associated with suicidal behavior risk. More broadly, the association between perceived burdensomeness and suicidal behavior risk appeared to be more pronounced at mean and high levels of negative emotional reactivity than at low levels.

One possible interpretation is that individuals with higher negative emotional reactivity may experience burden-related self-evaluations as more emotionally salient and persistent. Because negative emotional reactivity reflects a tendency for negative emotions to be more easily activated, more intense, and more prolonged, perceptions of being a burden on others may be accompanied by stronger affective responses among individuals with higher levels of this vulnerability. In this context, beliefs that one makes others' lives more difficult or imposes strain on significant others may be more closely linked to suicidal behavior risk. Accordingly, the present findings indicate that childhood trauma was associated with suicidal behavior risk both directly and conditionally indirectly through perceived burdensomeness, particularly when negative emotional reactivity was at average or elevated levels. Thus, perceived burdensomeness did not fully explain this association but appeared to represent one relevant interpersonal mechanism alongside a remaining direct association. Although studies directly examining perceived burdensomeness in relation to negative emotional reactivity remain limited, research linking emotional reactivity to suicidal ideation, suicide attempts, and suicidal behavior risk provides indirect support for the present findings (DeCou and Lynch, 2019; Shapero et al., 2019; Wang et al., 2021; Wu et al., 2023; Yang et al., 2023).

These findings are compatible with broader theoretical perspectives emphasizing emotional vulnerability in the context of adverse experiences and interpersonal stressors. For example, Linehan's biosocial model highlights emotional vulnerability and emotion regulation difficulties, particularly in adverse interpersonal contexts (Linehan, 2015). Similarly, vulnerability-stress perspectives and the Integrated Motivational–Volitional model suggest that early adverse experiences, individual vulnerability characteristics, and interpersonal processes may jointly be relevant to suicide-related risk (O'Connor and Kirtley, 2018). From this perspective, childhood trauma can be conceptualized as an early vulnerability context, whereas negative emotional reactivity may represent an affective context in which burden-related interpersonal self-evaluations are more strongly associated with suicidal behavior risk. However, because the present study did not directly test these broader theoretical models, these interpretations should be regarded as complementary rather than definitive explanations.

### 4.4 Limitations and future directions

Several limitations should be noted. First, because the study used a cross-sectional design, the findings should be interpreted as associations and conditional indirect associations rather than as evidence of causal or temporal pathways. Second, the sample consisted predominantly of women and young adults from a community sample, which may limit the generalizability of the findings to men, older adults, clinical populations, and individuals with more severe or complex trauma histories. Third, childhood trauma was assessed retrospectively and represented by a total score. Therefore, the present study could not examine whether the observed associations differ across specific forms of maltreatment, such as emotional abuse, physical abuse, sexual abuse, emotional neglect, and physical neglect, or according to contextual characteristics such as severity, chronicity, timing, and the relational context of trauma exposure. This issue is important because previous meta-analytic work suggests that the strength of associations with suicidal ideation and suicide attempts may vary across childhood maltreatment subtypes (Angelakis et al., 2019, 2020; Liu et al., 2017). Fourth, all variables were assessed using self-report measures, which may have introduced recall bias, social desirability bias, and shared method variance. Fifth, although the study was grounded in the IPTS, acquired capability for suicide was not assessed; therefore, the findings pertain to the interpersonal components of the theory rather than to the full IPTS model. Sixth, the study focused only on negative emotional reactivity and did not examine positive emotional reactivity, which may also be relevant to suicidal behavior risk. Finally, perceived burdensomeness and thwarted belongingness are conceptually related constructs, and findings from the simultaneous model should therefore be interpreted in terms of their unique associations after accounting for shared variance. In addition, as support resources were provided only at the end of the survey, future online studies on suicide-related topics may benefit from making these resources available from the outset and throughout participation.

Future research should examine whether the present pattern of findings is replicated in more diverse, clinical, and high-risk samples. In particular, longitudinal or prospective designs would help clarify the temporal sequence among childhood trauma, perceived burdensomeness, negative emotional reactivity, and suicidal behavior risk, as well as whether these interpersonal and emotional factors predict subsequent changes in suicide-related risk. Given the specific relevance of childhood maltreatment, further studies should also examine whether associations with perceived burdensomeness, thwarted belongingness, negative emotional reactivity, and suicidal behavior risk differ across trauma subtypes and according to severity, chronicity, timing, and relational context. In addition, future work may benefit from assessing acquired capability for suicide using validated measures such as the Acquired Capability for Suicide Scale–Fearlessness About Death (Ribeiro et al., 2014), and related interpersonal and emotional processes, including emotion regulation and interpersonal emotion regulation. Future research should use stratified or quota-based recruitment strategies to obtain more balanced representation across young, middle-aged, and older adult groups. Another important direction is to examine positive emotional reactivity as a potential protective factor, including whether it buffers the associations among childhood trauma, interpersonal processes, and suicidal behavior risk. Such research may help clarify whether perceived burdensomeness consistently shows a more specific association with suicidal behavior risk than thwarted belongingness when both interpersonal constructs are considered simultaneously, and whether negative emotional reactivity reliably marks a context in which burden-related self-evaluations are more strongly associated with suicidal behavior risk. In applied terms, the present findings may inform pilot prevention or intervention studies efforts for individuals with childhood trauma histories may benefit from targeting perceived burdensomeness, interpersonal connectedness, and emotion regulation capacities.

## 5 Conclusions

This study contributes to the literature by examining childhood trauma, the interpersonal components of the IPTS, negative emotional reactivity, and suicidal behavior risk within a single conditional process model. Perceived burdensomeness emerged as a more specific interpersonal correlate of suicidal behavior risk when perceived burdensomeness and thwarted belongingness were examined simultaneously. The conditional indirect association involving perceived burdensomeness was significant only at mean and high levels of negative emotional reactivity.

These findings suggest that perceived burdensomeness may be particularly important to consider when examining suicide-related risk in the context of childhood trauma, especially when elevated negative emotional reactivity is also present. Assessing themes such as feeling like a burden, believing that others would be better off in one's absence, and experiencing intense and persistent negative emotions may contribute to a more comprehensive risk formulation. In clinical contexts, these findings may support attention to dysfunctional interpersonal appraisals, social support, safety planning where clinically indicated, and emotion regulation skills for intense and rapidly escalating negative emotions. From a health-promotion perspective, these findings suggest that trauma-informed strategies in community settings may benefit from addressing interpersonal self-evaluations, strengthening social connectedness, and promoting adaptive emotion regulation skills for managing intense negative emotions.

In conclusion, the findings suggest that childhood trauma is associated with suicidal behavior risk in relation to interpersonal vulnerability processes, particularly perceived burdensomeness, and that this association may be more pronounced among individuals with higher negative emotional reactivity. These results highlight the importance of considering both interpersonal self-evaluations and emotional reactivity when examining suicide-related risk in the context of childhood trauma.

## Data Availability

The raw data supporting the conclusions of this article will be made available by the authors, without undue reservation.
